# The clinicopathological significance of FHIT hypermethylation in non-small cell lung cancer, a meta-analysis and literature review

**DOI:** 10.1038/srep19303

**Published:** 2016-01-22

**Authors:** Wei Yan, Ning Xu, Xiang Han, Xiao-ming Zhou, Bei He

**Affiliations:** 1 Department of Respiratory Medicine, Peking University Third Hospital, Beijing, 100191, China; 2 Department of Rheumatology, Peking University Third Hospital, Beijing, 100191, China; 3 Department of Respiratory Medicine, The Shengjing Hospital of China Medical University, Shenyang, 110004, China

## Abstract

Emerging evidence indicates that FHIT is a candidate tumor suppressor in non-small cell lung cancer (NSCLC). However, the correlation between *FHIT* hypermethylation and clinicopathological characteristics of NSCLC remains unclear. Thus, we conducted a meta-analysis to quantitatively evaluate the effects of *FHIT* hypermethylation on the incidence of NSCLC and clinicopathological characteristics. Final analysis of 1717 NSCLC patients from 16 eligible studies was performed. *FHIT* hypermethylation was found to be significantly higher in NSCLC than in normal lung tissue, the pooled OR from 8 studies including 735 NSCLC and 708 normal lung tissue, OR = 5.45, 95% CI = 2.15–13.79, *p* = 0.0003. *FHIT* hypermethylation was also correlated with sex status, smoking status, as well as pathological types. We did not find that *FHIT* hypermethylation was correlated with the differentiated types or clinical stages in NSCLC patients. However, patients with *FHIT* hypermethylation had a lower survival rate than those without, HR = 1.73, 95% CI = 1.10–2.71, *p*  = 0.02. The results of this meta-analysis suggest that *FHIT* hypermethylation is associated with an increased risk and worsen survival in NSCLC patients. *FHIT* hypermethylation, which induces the inactivation of *FHIT* gene, plays an important role in the carcinogenesis and clinical outcome and may serve as a potential drug target of NSCLC.

Lung cancer is the most frequent cause of cancer-related death in many countries, including China^1^,^2^. Lung cancers consist of two major histological types, small cell lung carcinoma (SCLC) and non-small cell lung carcinoma (NSCLC); the latter consists of squamous cell carcinoma (SCC), adenocarcinoma (AC), large cell carcinoma and others. NSCLC accounts for approximately 85% of all lung cancers, and there are approximately 80% of NSCLC cases in advanced stage where the prognosis remains poor^3^. Therefore, investigation of the mechanism of initiation, progression, and identification of prognostic markers is still needed for the selection of patients with NSCLC in order to provide better individualized treatment. Epigenetic modification of gene expression plays an important role in carcinogenesis. Aberrant methylation of CpG dinucleotides is a commonly observed epigenetic modification in human cancer^4^,^5^,^6^. Thus, the analysis of specific gene promoter methylation as a tool for diagnosis of tumors or its use as prognostic marker has been widely used for many different cancers including NSCLC^7^.

Fragile histidine triad protein (FHIT), also known as Bis (5′-adenosyl)-triphosphatase, is one of the histidine triad gene family members and is an enzyme encoded by the *FHIT* gene^8^,^9^. Previous reports showed that FHIT was inactivated by the loss of heterozygosity and methylation in cancer cells, which indicated that FHIT is a tumor suppressor protein^10^,^11^. Its precise function has been intensively studied in several tumors by the upregulation of inducing cell cycle arrest, apoptosis, inhibition of cell proliferation and by increasing its sensitivity to DNA damaging agents^12^,^13^,^14^. Lack of protein expression of *FHIT* by promoter methylation (hypermethylation) has been found to play an important role in lung alveolar differentiation regulation and epithelial tumorigenesis^15^,^16^,^17^,^18^. Although previous studies indicated that inactivation of the *FHIT* is mainly induced by hypermethylation of *FHIT* gene, the reported rates of *FHIT* hypermethylation in NSCLC were remarkably diverse. Moreover, whether it is associated with the incidence and clinical characteristics of NSCLC remains unclear. The variety of the study results underpin the need for assessing the evidence of the relationship between *FHIT* inactivation and NSCLC. Hence, we conducted a systematic review and meta-analysis to quantitatively evaluate the effects of *FHIT* hypermethylation on the incidence and clinical characteristics of NSCLC.

## Results

### Identification of relevant studies

**Figure 1:**
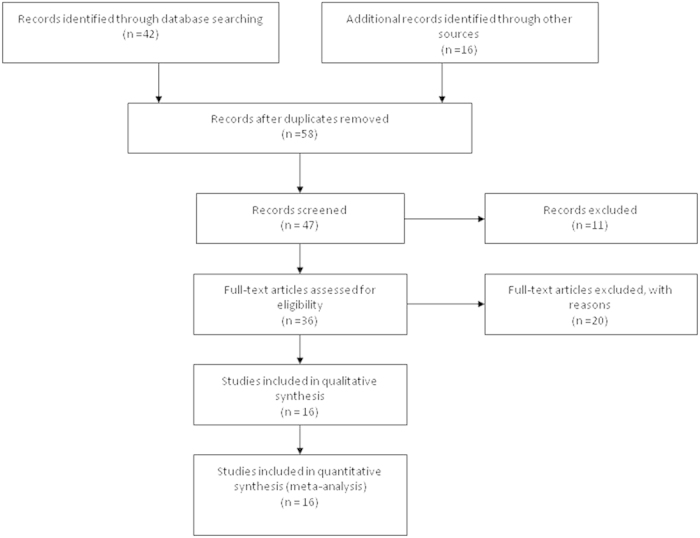
Flow chart of study selection.

Fifty eight publications were identified by the search method as described above. Forty two of those were excluded due to laboratory studies, non-original articles (review), or studies irrelevant to the current analysis. Eventually, there were sixteen studies included in final meta-analysis^16^,^19^,^20^,^21^,^22^,^23^,^24^,^25^,^26^,^27^,^28^,^29^,^30^,^31^,^32^,^33^ as shown in Fig. 1. We used Cohen’s kappa statistic to measure the agreement in the most important step for selecting eligible studies between two researchers and showed kappa value 0.76, indicating substantial observer agreement. Of [Fig f2]the sixteen studies, ten scored 8 points, six scored 7 points. Hence, the studies were of a relatively high quality (Table 1).

### Study characteristics

Sixteen studies published from 2001 to 2011 were eligible for meta-analysis. A total of 1717 NSCLC patients from China, South Korea, Japan, Italy, and USA was enrolled. Their basic characteristics are [Fig f3][Fig f4][Fig f5][Fig f6][Fig f7]summarized in Table 2.

### The correlation of FHIT hypermethylation with clinicopathological features

**Figure 2 f2:**
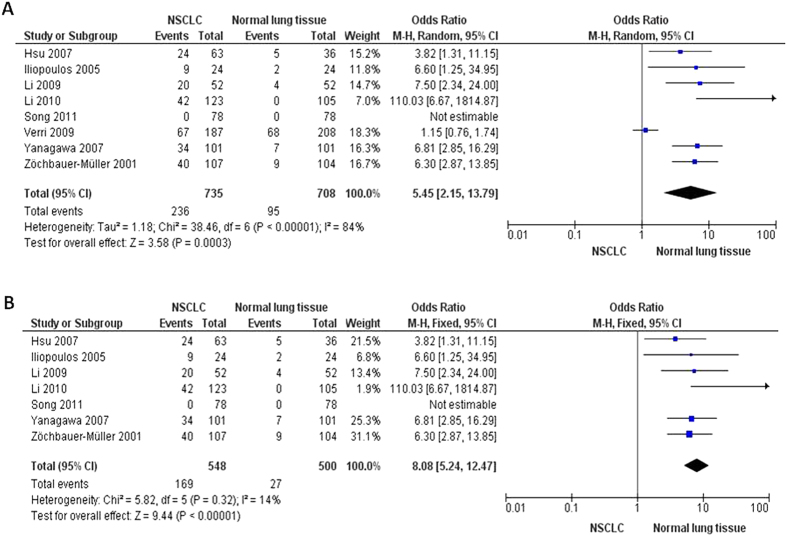
The pooled OR from 8 studies included 735 NSCLC and 708 normal lung tissues, I2 = 84%; OR = 5.45, 95% CI = 2.15–13.79, p = 0.0003. (**A**) The pooled OR from 7 studies included 548 NSCLC and 500 normal lung tissues, I^2^ = 14%; OR = 8.08, 95% CI = 5.24–12.47, *p* < 0.00001 (**B**).

**Figure 3 f3:**
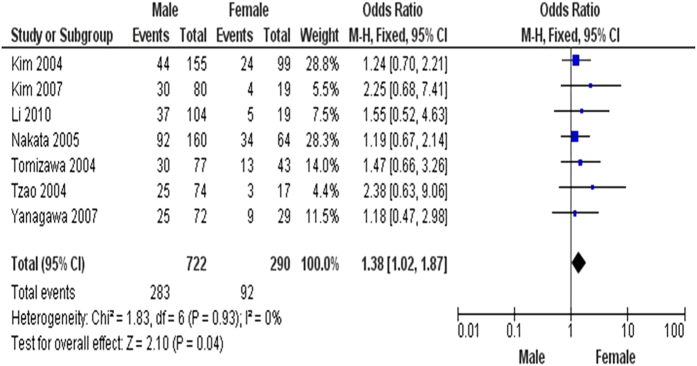
The pooled OR from 7 studies included 722 males and 290 females’ NSCLC, OR = 1.38, 95% CI = 1.02–1.87, p = 0.04, which indicates that FHIT hypermethylation is correlated with sex status in NSCLC patients.

**Figure 4 f4:**
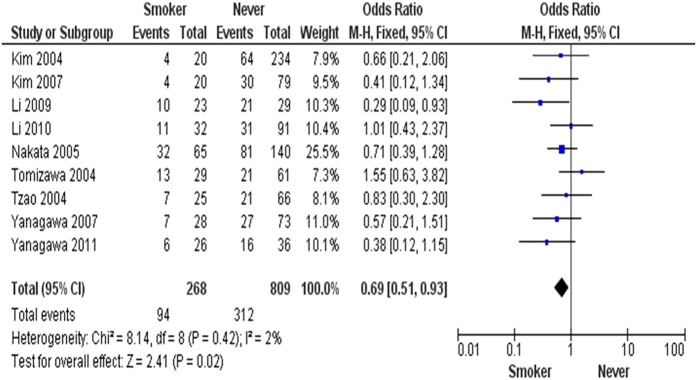
1077 NSCLC patients with the smoking status pooled in 9 studies. Aberrant *FHIT* hypermethylation is correlated with the smoking status in NSCLC patients, =0.69, 95% CI = 0.51–0.93, *p* = 0.02.

**Figure 5 f5:**
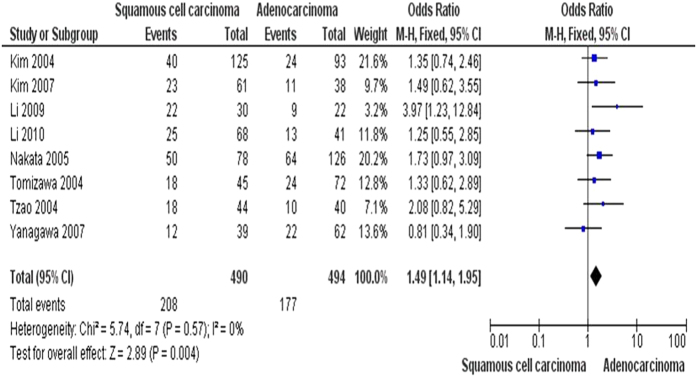
The pooled OR from 8 studies included 490 squamous cell carcinoma (SCC) and 494 adenocarcinoma (AD), OR = 1.49, 95% CI = 1.14–1.95, p = 0.004, indicates that FHIT hypermethylation plays more important role in the pathogenesis of SCC.

**Figure 6 f6:**
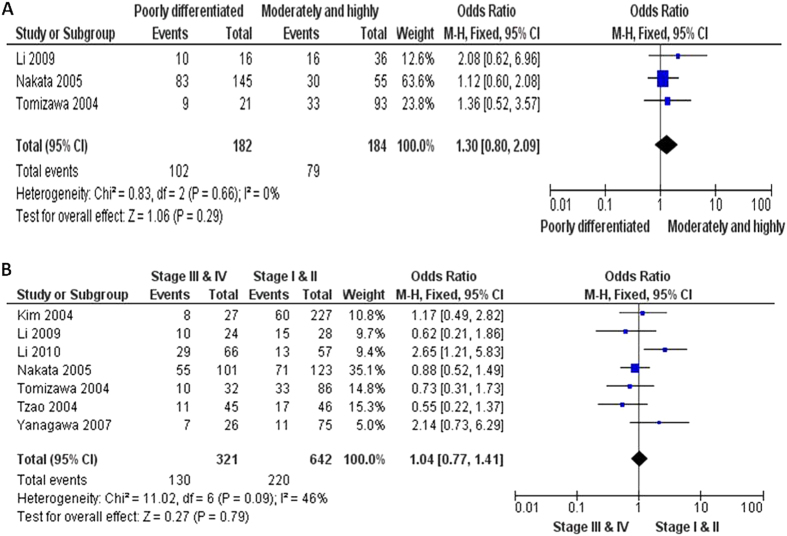
366 NSCLC patients were pooled from 3 studies to assess whether the aberrant FHIT hypermethylation in NSCLC was associated the differentiated status. Aberrant *FHIT* hypermethylation is not significantly higher in poorly differentiated NSCLC than that in moderately and highly differentiated NSCLC, OR = 1.30, 95% CI = 0.80–2.09, *p* = 0.29 (**A**). Aberrant *FHIT* hypermethylation is also not significantly higher in advanced NSCLC (III & IV) than that in early staged NSCLC (I & II), OR = 1.04, 95% CI = 0.77–1.41, *p* = 0.79 (**B**).

**Figure 7 f7:**
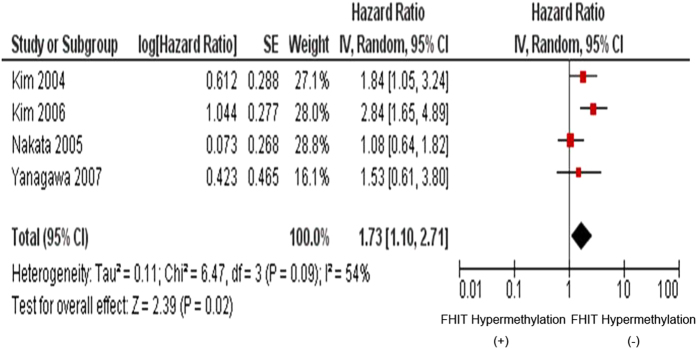
Four studies included were investigated for the relationship between overall survival (OS) and FHIT hypermethylation. The pooled HR for OS shows that *FHIT* hypermethylation is associated with worse survival in NSCLC (HR = 1.73, 95% CI = 1.10–2.71, *p* = 0.02).

1. The inactivation of *FHIT* through hypermethylation in NSCLC.We first determined that *FHIT* hypermethylation was significantly higher in NSCLC than in normal lung tissues. The pooled OR from 8 studies including 735 NSCLC and 708 normal lung tissues, is shown in Fig. 2A (OR = 5.45, 95% CI = 2.15-13.79, *p* = 0.0003), indicating that *FHIT* inactivation through hypermethylation plays an important role in the carcinogenesis of NSCLC. Since the heterogeneity is very high (I^2^ =  84%), we deleted one study (Verri 2009)^16^, re-calculated the pooled OR from remaining 7 studies and shown in Fig. 2B. I^2^ dramatically reduced to 14%, indicating that the heterogeneity is very low.
2. Relationship between the frequency of *FHIT* hypermethylation and sex status.Next, we determined whether *FHIT* hypermethylation rate was correlated with sex status. The pooled OR from 7 studies included 722 males and 290 females’ NSCLC, as shown in Fig. 3 (OR = 1.38, 95% CI = 1.02-1.87, *p* = 0.04), that indicate that *FHIT* hypermethylation was correlated with sex status in which it is higher in male than in female.
3. Relationship between the frequency of *FHIT* hypermethylation and smoking status.Then, we determined whether *FHIT* hypermethylation rate was correlated with smoking status. The pooled OR from 9 studies including 268 and 809 NSCLC with and without smoking history is shown in Fig. 4 (OR = 0.69, 95% CI = 0.51–0.93, *p* = 0.02), indicates that *FHIT* hypermethylation is correlated with smoking status in NSCLC patients.
4. Relationship between the frequency of *FHIT* hypermethylation and pathological types.We also determined whether *FHIT* hypermethylation was correlated with pathological types. The pooled OR from 8 studies including 490 squamous cell carcinoma (SCC) and 494 adenocarcinoma (AD), is shown in Fig. 5 (OR = 1.49, 95% CI = 1.14–1.95, *p* = 0.004), which indicates that *FHIT* hypermethylation plays a more important role in the pathogenesis of SCC.
5. The role of *FHIT* hypermethylation in NSCLC progression.We analyzed 366 NSCLC patients pooled from 3 studies to assess whether the aberrant *FHIT* hypermethylation in NSCLC was associated with the differentiated status. As shown in Fig. 6A, aberrant *FHIT* hypermethylation is not significantly higher in poorly differentiated NSCLC than that in moderately or highly differentiated NSCLC, OR = 1.30, 95% CI = 0.80–2.09, *p* = 0.29. In addition, aberrant *FHIT* hypermethylation is also not significantly higher in advanced NSCLC (III & IV) than that in early staged NSCLC (I & II), OR = 1.04, 95% CI = 0.77–1.41, *p* = 0.79, Fig. 6B. These results suggest that *FHIT* hypermethylation may not play an important role in NSCLC progression and different stages.
6. *FHIT* hypermethylation as a prognostic factor for NSCLC.There are 4 studies estimating the relationship between *FHIT* hypermethylation and overall survival (OS) in NSCLC patients. The pooled HR for OS shows that *FHIT* hypermethylation is associated with worsen survival in NSCLC patients as shown in Fig. 7 (HR = 1.73, 95% CI = 1.10–2.71, *p*  = 0.02).
7. Sensitivity analyses and publication bias.

**Figure 8:**
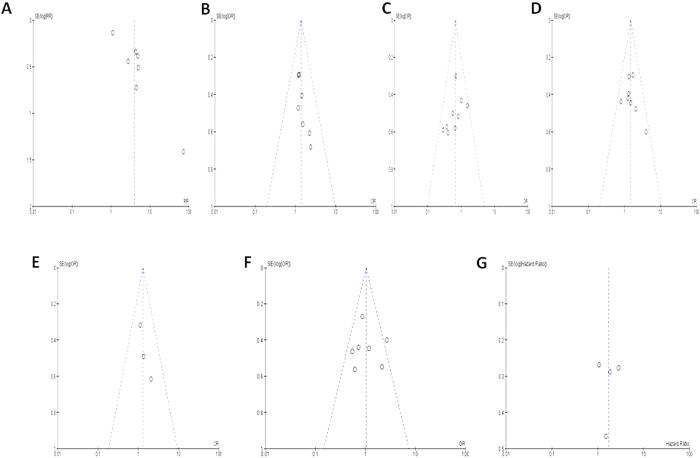
The funnel plots are largely symmetric, which suggests that there are no publication biases in the meta-analysis of FHIT hypermethylation and clinicopathological features. The funnel plot from 8 studies comparing NSCLC and normal lung tissue (**A**). The funnel plot from 7 studies determined the relationship between *FHIT* hypermethylation and the sex status in NSCLC patients (**B**). The funnel plot from 9 studies determined the relationship between *FHIT* hypermethylation and the smoking status in NSCLC patients (**C**). The funnel plot from 8 studies comparing *FHIT* hypermethylation between squamous cell carcinoma (SCC) and adnocarnoma (AD) (**D**). The funnel plot from 3 studies determined *FHIT* hypermethylation in different differentiated NSCLC (**E**). The funnel plot from 7 studies determined *FHIT* hypermethylation in different staged NSCLC (**F**). The funnel plot from 4 studies determined the relationship between *FHIT* hypermethylation and overall survival (OS) in NSCLC (**G**).

A sensitivity analysis, in which one study was removed at a time, was conducted to assess the result stability. In the case of relationship between *FHIT* hypermethylation in NSCLC and in normal lung tissue, the overall OR are in the range from 3.82 (95% CI: 1.31–11.15 to 110.03 (95% CI: 6.167–1814.87). The pooled ORs and HRs are not significantly changed, indicating the stability of our analyses. The funnel plots are largely symmetric, (Fig. 8A–G) suggesting there are no publication biases in the meta-analysis of *FHIT* hypermethylation and clinicopathological features.

## Discussion

Systematic reviews and meta-analyses have become increasingly important in biomedical science. Preferred Reporting Items for Systematic Reviews and Meta-Analyses (the PRISMA) are recommended to authorize the readers to access the strengths and weaknesses of the study.

### Interpretation of results and comparison with other studies

The *FHIT* gene locates the most common fragile site in the human genome, FRA3B (3p14.2), in which undergoes genomic rearrangement, biallelic loss, and cytogenetic abnormalities in tumors^8^,^34^,^35^. FHIT is genetically or epigentically altered in many primary and advanced carcinomas. Inactivation of *FHIT* by promoter hypermethylation plays an important role in tumorigenesis in several types of tumors including NSCLC^27^,^36^,^37^,^38^,^39^,^40^,^41^,^42^,^43^,^44^. To date, there have been some studies describing the methylation status of *FHIT* in NSCLC; however, the roles of methylation of *FHIT* in NSCLC and clinical significance have not been thoroughly investigated. We conducted the meta-analysis to determine the correlation between *FHIT* hypermethylation and clinicopathological characteristics in NSCLC. Analysis of the pooled data showed that (1) NSCLC has a higher hypermethylation than normal lung tissue; (2) *FHIT* hypermethylation is correlated with sex status in which it is higher in male than in female. (3) *FHIT* hypermethylation is correlated with smoking status in NSCLC patients. (4) *FHIT* hypermethylation is correlated with pathological types and plays a more important role in the pathogenesis of SCC. (5) *FHIT* hypermethylation is not significantly higher in poorly differentiated NSCLC than that in moderately or highly differentiated NSCLC. In addition, *FHIT* hypermethylation is also not significantly higher in advanced NSCLC (III & IV) than that in early staged NSCLC (I & II). (6) The pooled HR for OS shows that *FHIT* hypermethylation is associated with worsen survival in NSCLC patients. The cumulative evidence in our study is now conclusive that the *FHIT* gene promoter hypermethylation is associated with lung cancer formation and development, male gender, smoking behavior, and worse survival. In a meta-analyses of the gene methylation versus the cigarette smoking in NSCLC patients by Huang *et al*.^45^, *FHIT* methylation was found to be significantly associated with the smoking behavior, which support our conclusions. However, unavailability of meta-analysis or systemic review on other particular outcomes such as NSCLC initiation and development, gender and survival status makes it impossible to compare our results with other similar studies. The results suggest a potential role of *FHIT* methylation analysis in diagnosis and prognosis of lung cancer in clinical settings. Epigenetic alteration, particularly aberrant DNA methylation, is one of the best-characterized epigenetic modifications that contribute to tumor initiation and progression^5^,^6^. FHIT is thought to affect cellular function and behavior largely through its signaling properties. FHIT also activates caspase-8 and caspase-2, which causes the release of cytochrome c and finally induces apoptosis^46^. FHIT and p53, the two most commonly altered tumor suppressor genes, might rely on common mediators and crosstalk among these proteins in regulation of growth-related pathways; thus, the inactivation of both genes results in prominent deregulation of cell proliferation and tumor progression in lung cancer^47^. Huang *et al*. showed that 7 hypermethylated genes including *FHIT* were significantly associated with the smoking behavior in NSCLC patients^45^. The difference about the result may be due to the different selected number of studies. They selected only 5 studied which included 518 patients. Our studies searched 9 studies which included 1077 patients. A number of studies showed that inactivation of FHIT can cause tumor aberrant progression and link to clinicopathological characteristics^27^,^48^,^49^,^50^,^51^. Therefore, FHIT can be considered as a tumor suppressor, and its inactivation could contribute tumor progression and poor prognosis. Although only four studies evaluated the relationship between overall survival and *FHIT* hypermethylation in NSCLC, they showed very similar results^25^,^27^,^28^,^32^. Based on this meta-analysis, the pooled HR for OS showed that *FHIT* hypermethylation was associated with worsen survival in NSCLC patients, HR = 1.73, 95% CI = 1.10–2.71, *p*  = 0.02. Therefore, we may consider that *FHIT* hypermethylation in NSCLC tends to indicate a poor prognosis.

### Strengths and limitations of evidence

In the comparison cancer and normal lung tissue, the heterogeneity is very high (I^2^ = 84%), thus we deleted one study (Verri 2009)^16^, re-calculated the pooled OR from remaining 7 studies and shown in Fig. 2B. I^2^ dramatically reduced to 14%, indicating that the heterogeneity is very low. The reason of their results were total different from other studies is not clear, they could have used inappropriate primers and methylation specific PCR (MSP) condition in detection of *FHIT* hypermethylation.

Consistent results were shown in sensitivity analyses, and no evidence of publication bias was found. This study has several potential limitations. First, the possibility of information and selection biases and unidentified confounders could not be completely excluded because all of the included studies were observational. Second, the searching strategy was restricted to articles published in English. Articles with potentially high-quality data that were published in other languages were not included because of the anticipated difficulties in obtaining accurate medical translation. Hence, cautions should be taken when our findings are interpreted among the general populations.

### Research and Clinical implications

The results from the current study demonstrate that the hypermethylation rate of *FHIT* gene promoter in NSCLC is significantly higher than that in the normal lung tissues, indicating that *FHIT* promoter hypermethylation is common in NSCLC. Since changes in *FHIT* promoter hypermethylation are reversible, drug treatment through demethylation may be useful to delay carcinogenesis and progression and to improve prognosis. Lung cancer cell clones carrying conditional FHIT transgenes showed significant suppression of xenograft tumor growth, suggesting that treatments to restore endogenous FHIT expression in lung cancers would result in decreased tumorigenicity^17^. In addition, injection of 5-aza-2-deoxycytidine (AZA) and trichostatin A (TSA) in nude mice with established H1299 tumors showed suppressed growth of small tumors without apparent toxicity, and responding tumors showed restoration of FHIT^17^. These preclinical studies show the therapeutic potential of restoration of tumor suppressor expression through epigenetic modulation. This approach may bring new direction and hope for cancer treatment through gene-targeted therapy.

In conclusion, our meta-analysis shows that NSCLC had a higher *FHIT* hypermethylation than normal lung tissue, higher in male than in female, higher in non-smoker than in smoker, and higher in SCC than in AD. In addition, *FHIT* hypermethylation is associated with an increased risk and worsen survival in NSCLC. Further large-scale studies, especially multi-center and well-matched cohort research, will provide more insight into the role of *FHIT* in the prognosis and clinical implementation of NSCLC patients.

## Material and Methods

### Information sources

#### Key database searched, data extraction and methodological assessment

We searched Pubmed, Embase, and ISI web of knowledge to identify studies from May 1, 1998 to March 1, 2014 using the search terms: “lung” and “cancer or tumor or neoplasm or carcinoma”, “methylation”, and “FHIT or Fragile histidine triad protein or Bis (5′-adenosyl)-triphosphatase”. We also searched manually for the reference lists of the retrieved articles and reviews for additional articles.

Although our search did not have language limits initially, for the full-text reading and final evaluation we only performed the review of the studies published in English language. After excluding non-relevant and/or redundant publications from different databases, the remaining papers were evaluated in the full text version for in- and exclusion criteria and for relevant articles in the reference lists. All searched data were retrieved. Authors’ bibliographies and references of selected studies were also searched for other relevant studies. The most complete study was chosen to avoid duplication if same patient populations were reported in several publications.

Two authors (WY, NX) independently reviewed and extracted data from eligible studies. Disagreements were resolved by discussion and consensus with a third author (BH). The following information was recorded for each study: the first author name, year of publication, sample source, number of cases, clinicopathological parameters, cancer TNM (tumor node metastasis) stage, methylation detection method, methylation rate and/or expression, and follow up. Data for study characteristics and clinical responses were summarized and organized into a table format. Heterogeneity of investigation was evaluated to determine whether the data of the various studies could be analyzed for a meta-analysis.

For the methodological evaluation of the studies, three investigators (XZ, XH and BH) read through each publication independently, and they assessed and scored studies according to the Newcastle-Ottawa Scale (NOS)^52^, which was developed to assess the quality of nonrandomised studies with its design, content and ease of use directed to the task of incorporating the quality assessments in the interpretation of our meta-analytic results (Table 1). The three readers provided the quality scores and compared them, and then they reached a consensus value for each item.

### Eligibility criteria

Criteria that an eligible study has to meet were as follows: (1) *FHIT* hypermethylation evaluated in the primary NSCLC tissues, (2) researches revealed the relationship between *FHIT* hypermethylation and NSCLC clinicopathological parameters and prognosis, (3) *FHIT* hypermethylation examined by polymerase chain reaction (PCR), (4) studies provided sufficient information to estimate hazard ratio (HR) about overall survival (OS) and 95% confidence interval (CI). The exclusion criteria included the following: (1) letters, reviews, case reports, conference abstracts, editorials, expert opinion, (2) all publications regarding *in vitro*/*ex vivo* studies, cell lines and human xenografts were also excluded.

### Risk of bias

Publication bias was assessed by using a method reported by Egger *et al*.^53^. We also explored reasons for statistical heterogeneity using meta-regression, subgroup analysis, and sensitivity analysis. The analysis of meta-regression and publication bias was performed by using STATA version 10.0. Cohen’s kappa statistic was used to measure the agreement among the most important step for selecting eligible studies between two researchers. The kappa values were interpreted as follows: <0.2, poor observer agreement; 0.2–0.4, fair observer agreement; 0.4–0.6, moderate observer agreement; 0.6–0.8, substantial observer agreement; and 0.8–1.0, good observer agreement^54^.

Statistical analysis: Analysis was conducted using the STATA 12.0 (Stata Corporation, TX, USA) and Review Manager 5.2 (Cochrane Collaboration, Oxford, UK). The pooled frequency of *FHIT* hypermethylation and 95% confidence intervals (95% CI) were estimated. The frequency of *FHIT* hypermethylation was compared in different tumor characteristics. Heterogeneity among studies was evaluated with Cochran’s Q test^55^ and the *I*^*2*^ statistic^56^,^57^. When heterogeneity was not an issue (*I*^*2*^ values <50%), a fixed effect model was used to calculate parameters. If there was substantial heterogeneity (*I*^*2*^ values ≥50%), a random-effects model was used to pool data and attempt to identify potential sources of heterogeneity based on subgroup analyses. The pooled OR was estimated for the association between *FHIT* hypermethylation and clinicopathological features. *P* values tailed less than 0.05 were considered statistically significant.

## Additional Information

**How to cite this article**: Yan, W. *et al*. The clinicopathological significance of FHIT hypermethylation in non-small cell lung cancer, a meta-analysis and literature review. *Sci. Rep.* **6**, 19303; doi: 10.1038/srep19303 (2016).

## Figures and Tables

**Table 1:** Quality assessment according to the Newcastle-Ottawa scale of the included studies.

Study	Language	Selection	Comparability	Exposure	Totalscore
Zöchbauer-Müller, *et al*. 2001^19^	English	3	2	3	8
Hsu, *et al*. 2007^20^	English	3	2	3	8
Yanagawa, *et al*. 2011^21^	English	3	2	3	8
Song, *et al*. 2011^22^	Chinese	3	2	2	7
Li, *et al*. 2010^23^	English	3	2	3	8
Li, *et al*. 2009^24^	Chinese	3	2	2	7
Verri, *et al*. 2009^16^	English	3	2	3	8
Yanagawa, *et al*. 2007^25^	English	3	2	2	7
Kim, *et al*. 2007^26^	English	3	2	2	7
Kim, *et al*. 2006^27^	English	3	2	3	8
Nakata, *et al*. 2005^28^	English	3	2	3	8
Iliopoulos, *et al*. 2005^29^	English	3	2	3	8
Tomizawa, *et al*. 2004^30^	English	3	1	3	7
Tzao, *et al*. 2004^31^	English	3	2	3	8
Kim, *et al*. 2004^32^	English	3	2	2	7
Maruyama, *et al*. 2004^33^	English	3	2	3	8

**Table 2:** Basic characteristics of the included studies.

Study	Country	Patients	Methods	Primary Aim	Methylation site	FHIT expression
Zöchbauer-Müller, *et al*. 2001^19^	United States	107	Methylation specific PCR (MSP)/Northern blot analysis	Determine the correlation of protein and hypermethylation status of *FHIT* in lung and breast cancer	Promoter, CpG islands	+
Hsu, *et al*. 2007^20^	China	63	MSP	Determine the frequency of six genes’ hypermethylation in NSCLC	Promoter, CpG islands	−
Yanagawa, *et al*. 2011^21^	Japan	62	MSP	Determine the methylation status of Multiple genes in NSCLC	Promoter, CpG islands	−
Song, *et al*. 2011^22^	China	78	MSP/RT-PCR	Aims to determine the methylation status of five tumor suppressor in NSCLC	Promoter, CpG islands	+
Li, *et al*. 2010^23^	China	123	MSP	Determine the methylation status of *FHIT* in NSCLC	Promoter, CpG islands	−
Li, *et al*. 2009^24^	China	52	MSP/RT-PCR	Explore the effects of CpG island methylation on protein and mRNA expression of FHIT in NSCLC	Promoter, CpG islands	+
Verri, *et al*. 2009^16^	Italy	187	MSP/Immuno- histochemistry	Determine the inactivation of *FHIT* in NSCLC	Promoter, CpG islands	+
Yanagawa, *et al*. 2007^25^	Japan	101	MSP	Determine the methylation status of ten genes in pathogenesis of NSCLC	Promoter, CpG islands	−
Kim, *et al*. 2007^26^	South Korea	99	MSP	Determine methylation patterns of eight tumor suppressor gene in NSCLC	Promoter, CpG islands	−
Kim, *et al*. 2006^27^	South Korea	335	MSP	The methylation profile of 5 genes for NSCLC were analyzed and correlated with clinical data	Promoter, CpG islands	−
Nakata, *et al*. 2005^28^	Japan	139	MSP/Immuno- histochemistry	Determine the inactivation of *CDH1*, *p16* and *FHIT* in NSCLC	Promoter, CpG islands	+
Iliopoulos, *et al*. 2005^29^	United States	24	MSP/Immuno- histochemistry	Determine the inactivation of *FHIT* and *WWOX* in lung, breast and bladder cancer	Promoter, CpG islands	+
Tomizawa, *et al*. 2004^30^	Japan	54	MSP	Investigate the clinicopathological significance of aberrant methylation of *RARβ*2, *RASSF1A* and *FHIT* in NSCLC patients	Promoter, CpG islands	−
Tzao, *et al*. 2004^31^	China	44	MSP/RT-PCR	Examines protein, mRNA expression, and hypermethylation of the *FHIT* gene in NSCLC	Promoter, CpG islands	+
Kim, *et al*. 2004^32^	South Korea	125	MSP	Determine the clinicopathological and prognostic significance of *FHIT* methylation in NSCLC	Promoter, CpG islands	−
Maruyama, *et al*. 2004^33^	United States	124	MSP	Determine the correlation between the aberrant promoter methylation of multiple genes and survival in patients with NSCLC	Promoter, CpG islands	−
